# The Microstructure and Pitting Corrosion Behavior of K-TIG Welded Joints of the UNS S32101 Duplex Stainless Steel

**DOI:** 10.3390/ma16010250

**Published:** 2022-12-27

**Authors:** Shuwan Cui, Shuwen Pang, Dangqing Pang, Fuyuan Tian, Yunhe Yu

**Affiliations:** 1 School of Mechanical and Automotive Engineering, Guangxi University of Science and Technology, Liuzhou 545006, China; 2 Guangxi Zhuang Autonomous Region Tobacco Company Liuzhou Tobacco Company, Liuzhou 545006, China

**Keywords:** K-TIG welding, microstructure, pitting corrosion resistance, E_p_ value, chromium nitride precipitates

## Abstract

In this paper, the microstructure and pitting corrosion resistance of S32101 duplex stainless steel keyhole tungsten inert gas welded joints with different heat inputs were studied. The electrochemical experiments were conducted in a 1 mol/L NaCl solution at room temperature. The pitting rupture potential of the heat affected zone and the weld metal zone under different heat inputs were tested. The research showed that with the increase of heat inputs, more ferrite was converted to austenite and the number and size of intragranular austenite grains in the weld metal zone increased. The austenite content of the heat affected zone and the weld metal zone increase with the increase of heat inputs, and the CrN and Cr_2_N in the heat affected zone and the weld metal zone were mainly precipitated in the ferrite, in the austenite and ferrite/austenite interfaces. The pitting rupture potential value of the heat affected zone and the weld metal zone were increased with the increase of heat inputs, and the pitting corrosion resistance of the heat affected zone and weld metal zone were also increased with the increase of heat inputs. The relationship between the position CrN and Cr_2_N, the austenite content and the pitting corrosion resistance were elucidated, and the initiation mechanism of the pitting was investigated. Additionally, in this work, the heat affected zone and weld metal zone made at 2.46 kJ/mm heat inputs had the best pitting corrosion resistance. The research results provided useful information for improving the pitting corrosion resistance of S32101 duplex stainless steel keyhole tungsten inert gas welded joints.

## 1. Introduction

Duplex stainless steel (DSS) belongs to a corrosion-resistant alloy, which was consists of a ferrite phase of the body-centered cubic structure and an austenite phase of the face-centered cubic structure, and has the advantages of a great stress corrosion resistance, a good toughness and a high strength [1,2]. The traditional welding method is easy to destroy the reasonable two-phase structure of the DSS, causing the decrease of mechanical properties and the corrosion resistance of the weld joints (WJs). In particular, the DSS is used in corrosive environments, such as marine construction, seawater equipment and various types of chemical plants. With uneven microstructure, it is very easy to cause the pitting and failure of the WJs [3,4,5,6,7]. A novel and effective welding method has therefore some significance for the reliability and safety of DSS products.

Keyhole tungsten inert gas (K-TIG) welding is a type of high-current keyhole welding method, which can generate a high-energy and high-stiffness arc, to achieve a large penetration depth [1,3,8]. K-TIG welding can form high-quality, high-performance WJs without the need for complex pre-welding treatments. However, WJs inevitably produce some harmful phases after undergoing welding thermal cycles, including carbides, sigma phase, chi phase and chromium nitride [9,10,11,12]. These undesired precipitate phases can cause the mechanical properties and corrosion resistance of WJs to deteriorate dramatically [3,13]. Most of the current research is based on the study of the microstructure of the mechanical properties of WJs, and the corrosion behavior has received little attention so far. 

Once pitting corrosion is initiated, it can continue to grow, leading to structural damage [7,14]. Consequently, it is recognized as a harmful form of corrosion. Several reports [15,16,17,18] have proved that the precipitation of harmful precipitates in the matrix, such as chromium carbide or chromium nitride, can destroy the continuity of the passivation film of the matrix, resulting in the deterioration of the pitting corrosion resistance of the WJs, which is recognized as the initiation mechanism of pitting corrosion. Kumar and Köse et al. [19,20] had indicated that the unreasonable HI could lead to a phase ratio (ferrite/austenite) imbalance and the formation of chromium nitride. Shi et al. [21] found that HI could affect the precipitation amount of chromium nitride in the heat affected zone (HAZ) and the intergranular corrosion resistance of the HAZ. Consequently, the reasonable control of the welding heat inputs (HIs) could effectively improve the corrosion resistance of the WJs.

In this paper, the evolution of the microstructure, the austenite content, the chromium nitride precipitates and the initiation mechanism of the pitting corrosion of the S32101 DSS K-TIG WJs with HIs varying from 1.99–2.46 kJ/mm, were investigated. The research results provide useful information for improving the pitting corrosion resistance of S32101 DSS K-TIG WJs.

## 2. Experimental Procedure

### 2.1. Materials and Welding Procedure

**Figure 1:**
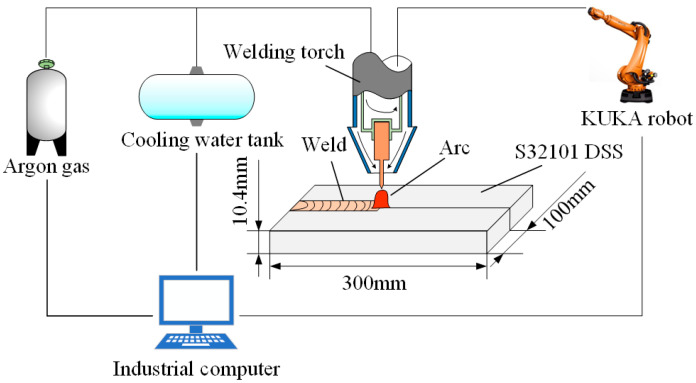
The experimental arrangement of the K-TIG welding.

The thickness of 10.4 mm S32101 DSS plates were selected as the base material (BM). The chemical compositions and dimensions of the BM are listed in Table 1 and Figure 1, respectively. Prior to clamping the fixture, the surface of the workpiece was ground using sand paper, and then, the workpieces were cleaned with acetone to remove any contamination and oxides on the surfaces. The butt welding was applied to ensure that the workpieces were held stationary with no gap between the workpiece and the fixture. High purity argon (99.9%) was continuously fed into the molten pool as a protective gas at a flow rate of 20 L/min in the welding process, ensuring the reliability and stability of the welding. The specific welding parameters are shown in Table 2. According to ASTM E1245-03 (2016), the optical microscope (OM) images of each specimen were captured and the content of austenite was calculated using Image-Pro Plus software (Media Cybernetics, Rockville, MD, USA). The austenite content of each sample was calculated from 10 different images, and the average value was taken as the final result. The distribution and quantity of CrN and Cr_2_N were revealed by EBSD (Oxford Instruments, Oxford, England). In each test, the specimens for the EBSD test were ground to 1000 grit and electrolytically polished in a 50 mL HClO_4_ + 650 ml C_2_H_5_OH + 100 ml H_2_O solution for 20 s. The configuration of the step size in the EBSD analysis was 3.5 μm. In order to ensure the accuracy of the data, the EBSD maps removed the wild spikes and performed the noise reduction. 

### 2.2. Electrochemical Experiments

**Figure 2:**
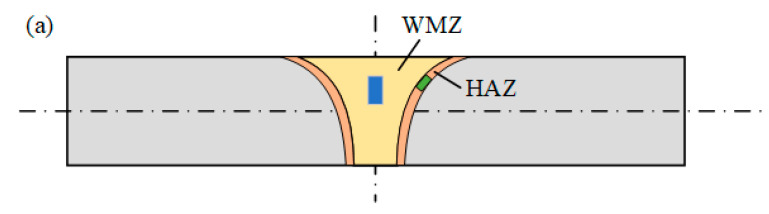

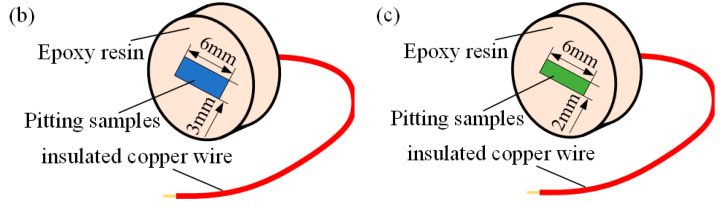
Schematic of the electrochemical test specimens. (**a**) Sampling location of the HAZ and WMZ; (**b**) The electrochemical test specimen of the WMZ and (**c**) The electrochemical test specimen of the HAZ.

The electrochemical experiments were conducted in a 1 mol/L NaCl solution at room temperature. A classic three-electrode system was utilized, based on the ASTM G150-99(2004) standard. A saturated calomel electrode (SCE) was set as the reference electrode and platinum (Pt) foil was used as the counter electrode. The working electrode was made by embedding the HAZ and the weld metal zone (WMZ) of the WJs with different HIs into epoxy resin, as shown in Figure 2. The polarization curve was measured with a scan rate of 1 mV/s. The area of the HAZ and WMZ was selected to compare the corrosion resistance of the two different regions of the WJs. Meanwhile, 704 silicone rubber was used to encapsulate the interface between the stainless steel sample and the epoxy resin for the protection of the crevice corrosion. At least two sets of measurements of each zone were carried out to ensure the reproducibility. The microstructure of the WJs after the electrochemical corrosion was observed by OM.

## 3. Results and Discussion

### 3.1. The Microstructure of the K-TIG WJs 

The microstructure of the K-TIG WJs under different HIs is displayed in Table 3. The microstructure of the HAZ and WMZ were composed of austenite (light phase) and ferrite (dark phase) phases, and the austenite mainly exists in the form of intragranular austenite (IGA), grain boundary austenite (GBA) and Widmanstätten austenite (WA). Under the action of the welding thermal cycle, a part of the austenite dissolved in ferrite, and the ferrite grain obviously coarsened. During the subsequent cooling process, the austenite was gradually precipitated from the ferrite. Higher HIs reduced the cooling rate during the welding [22,23,24], so the austenite in the HAZ had enough time to precipitate and the GBA also coarsened with the increase of the HIs. Compared with the traditional welding method, the K-TIG welding speed was relatively fast, so when the HI was relatively small, the coarse austenite was easy to form WA [3]. With the increase of the HI, the corresponding cooling time was relatively long, which further promoted the transformation of more ferrite into austenite. Therefore, the number and size of IGA grains in the WMZ also increased. 

### 3.2. The Austenite Content and Chromium Nitride Precipitates of the K-TIG WJs 

The austenite content and the chromium nitride precipitates of the HAZ and WMZ in the WJs under different His, are shown in Table 4. The experiment results show that the contents of austenite in the HAZ and WMZ increase with the increase of the HI. The content of austenite in the WMZ was higher than that in the HAZ under the same HI. Two kinds of nitride precipitates, CrN and Cr_2_N, were found in the HAZ and WMZ. No carbides and σ phases were detected, as has been proved in previous studies [3,21]. Table 4 shows the locally enlarged views of the nitride precipitates in the HAZ and WMZ. Two kinds of nitride precipitates, CrN and Cr_2_N, were detected in the HAZ and WMZ of the K-TIG WJs. The contents of CrN and Cr_2_N in the HAZ and WMZ measured by the EBSD software, decrease with the increase of the HI, which has been confirmed by previous studies [3,21]. When the HI was 2.46 kJ/mm, no chromium nitride precipitates were detected in the WMZ. As can be seen from Table 4, compared with Cr_2_N, CrN was more easily formed in the HAZ. It is mainly because the cooling rate of the HAZ was faster than that of the WMZ, and CrN was more easily formed when the cooling rate was higher than 100 K/s. In addition, the CrN and Cr_2_N in the HAZ and WMZ were mainly precipitated in the ferrite, in the austenite, and in ferrite/austenite interfaces.

### 3.3. Electrochemical Corrosion Properties

**Figure 3:**
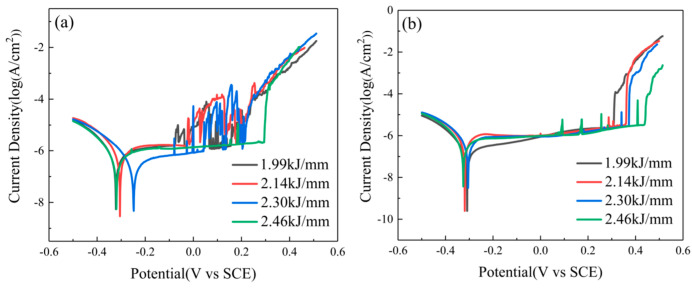
Polarization curves of the WJs in 1mol/L NaCl solution at room temperature: (**a**) HAZ and (**b**) WMZ.

Figure 3 shows the polarization curves of the WMZ and HAZ in the WJs under different HIs. All of the test specimens underwent a passivation state before the stable pitting occurred, that is, the current density basically remained unchanged as the potential rose. However, severe current fluctuations in the HAZ appeared before the appearance of the pitting rupture potential (E_P_) with the continuous increase of the potential, indicating that the metastable pitting occurs in the HAZ, as shown in Figure 3a. The supersaturated nitrogen (N) would combine with chromium (Cr) to form the precipitation phases of CrN and Cr_2_N, under the condition of the rapid cooling of the HAZ in the WJs [21]. Compared with the WMZ, more CrN and Cr_2_N were formed in the HAZ, which results in the destruction of the continuity of the passivation film. The metastable pitting corrosion phenomenon was mainly because the passivation film around the precipitation phases would be pitted first under the corrosion of the Cl^–^ ions, and when the passivation film in the etching pits was completely destroyed, the solution inside and outside the etching pits would rapidly mix, and the corrosiveness of the Cl^–^ ions would be reduced below a critical concentration, which made the etching pits passivate again [25,26,27]. Table 5 summarizes the electrochemical characteristics of the specimens obtained from the polarization curves analyzed by Tafel extrapolation, where E_corr_ is the corrosion voltage and I_corr_ is the corrosion current. The E_P_ of the HAZ and WMZ increased with the increase of the HI. Therefore, the pitting corrosion resistance of the HAZ and WMZ increased with the increase of the HI. Under the same HI condition, the value of E_P_ − E_corr_ in the HAZ was significantly lower than that of the WMZ, indicating that the WMZ had a more stable passive film. So the pitting resistance of the WMZ was superior to that of the HAZ, which was consistent with the conclusion of previous studies [28].

**Figure 4:**
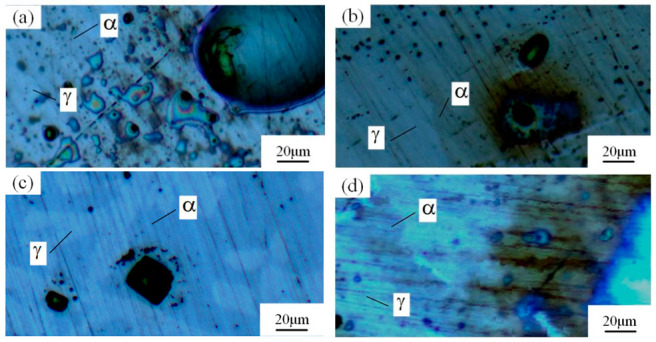
The microstructure of the HAZ after the pitting test under different HIs. (**a**) 1.99 kJ/mm (**b**) 2.14 kJ/mm (**c**) 2.30 kJ/mm and (**d**) 2.46 kJ/mm.

Chen and Zhang et al. [29,30] have demonstrated that the austenite had a higher pitting corrosion resistance than the ferrite. Figure 4 shows that the change law of the austenite content in the HAZ and WMZ of the WJs. The austenite content of the WMZ was significantly higher than that of the HAZ under the same HI. Therefore, the pitting corrosion resistance of the WMZ was greater than that of the HAZ, and the pitting corrosion resistance of the WJs was gradually enhanced with the increase of the HI which was consistent with the test results of the electrochemical corrosion experiments. From Table 4, it can be seen that the austenite content of the WMZ with a HI of 1.99 kJ/mm was lower than that of the HAZ with a HI of 2.46 kJ/mm, but the E_P_ value of the WMZ with a HI of 1.99 kJ/mm was greater than that of the HAZ with a HI of 2.46 kJ/mm. The results show that the pitting corrosion resistance of the test specimen did not increase with the increase of the austenite content, and the austenite content was not the only factor affecting the pitting corrosion resistance of the WJ. 

**Figure 5:**
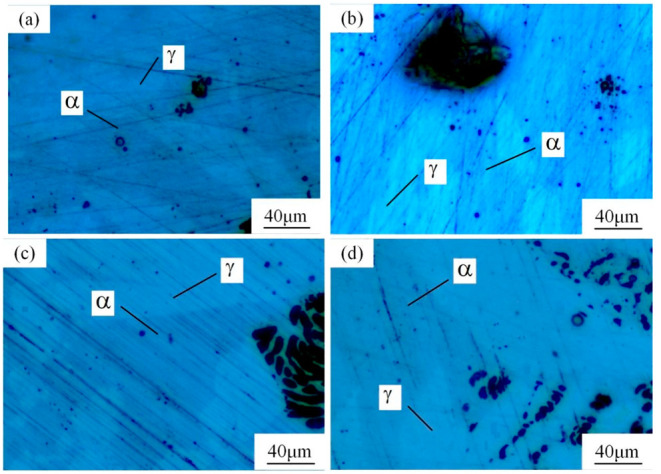
The microstructure of the WMZ after the pitting test under different HIs. (**a**) 1.99 kJ/mm (**b**) 2.14 kJ/mm (**c**) 2.30 kJ/mm and (**d**) 2.46 kJ/mm.

Many studies have confirmed that N could segregate anodically to the metal-passive film interface during the passivation, which stabilized the passive film and prevented the attack of the aggressive ${\mathrm{Cl}}^{-}$ ions [31,32]. The precipitated phases of CrN and Cr_2_N were precipitated during the welding cooling process due to the dramatic decrease of the N solubility, so the amount of the CrN and Cr_2_N precipitation greatly affected the pitting corrosion resistance of the WJs. The surface morphologies of the corroded specimens of the HAZ and WMZ under different HIs are presented in Figure 4 and Figure 5, showing the position of the pitting corrosion. Figure 4 and Figure 5 show that pitting occurs mainly in the ferrite and ferrite/austenite interface in both the HAZ and WMZ, and then propagated towards ferrite. It can be found from Table 4 that the chromium nitride precipitates were mainly distributed at the phase boundary between austenite and ferrite. The results showed that the pitting corrosion positions are the same as the positions of CrN and Cr_2_N. Thus it can inferred that the pitting occurred first in the vicinity of CrN and Cr_2_N and later expanded and grew in the surrounding area. This is mainly because the location of CrN and Cr_2_N destroyed the passivation film of the WJs, and the vicinity of CrN and Cr_2_N were first corroded by $the\;{\mathrm{Cl}}^{-}$ ions in the solution, resulting in the formation of cracks or cracks between the chromium nitride precipitates and the stainless steel ferrite matrix. As a result, the pitting occurred first in pits created by cracks or cracks, and then the pitting corrosion gradually extended to the ferrite area with a poor pitting resistance [29,30]. Therefore, it can be inferred that the pitting resistance of the K-TIG WJs decreased with the increase of the CrN and Cr_2_N precipitation.

## 4. Conclusions

(1) With the increase of the HI, more ferrite of the WJs was transformed into austenite and the number and size of IGA grains in the WMZ also increased.
(2) With the increase of the HI, the austenite content of the HAZ and WMZ increased. The CrN and Cr_2_N in the HAZ and WMZ were mainly precipitated in the ferrite, in the austenite and ferrite/austenite interfaces.
(3) The pitting corrosion resistance of the WMZ was greater than that of the HAZ, and the pitting corrosion resistance of the HAZ and WMZ improved with the increase of the HI.
(4) The pitting occurred first in the vicinity of the chromium nitride precipitates and later expanded and grew in the surrounding area. Meanwhile, the pitting corrosion resistance of the WJs was gradually weakened with the increase of the precipitation amount of CrN and Cr_2_N.

## Figures and Tables

**Table 1:** The chemical compositions of the S32101 DSS (wt.%).

Element	S	C	P	Cu	N	Mo	Si	Ni	Mn	Cr	Fe
S32101	0.002	0.017	0.02	0.16	0.21	0.22	0.49	1.56	4.98	21.52	70.821

**Table 2:** The specific welding parameters of the K-TIG welding.

Parameters	Levels
Welding power (W)	7755, 8330, 8925, 9540
Welding speed (mm/s)	3.5
Welding thermal efficiency	0.9
Heat input (kJ/mm)	1.99, 2.14, 2.30, 2.46

**Table 3:** The microstructure of the K-TIG WJs under different HIs about: (HAZ: (**a**) 1.99 kJ/mm; (**b**) 2.14 kJ/mm; (**c**) 2.30 kJ/mm and (**d**) 2.46 kJ/mm; WM: (**e**) 1.99 kJ/mm; (**f**) 2.14 kJ/mm; (**g**) 2.30 kJ/mm and (**h**) 2.46 kJ/mm. (Note: intragranular austenite (IGA), grain boundary austenite (GBA) and Widmanstätten austenite (WA)).

Heat input (kJ/mm)	Welded joint	
	HAZ	WM
1.99	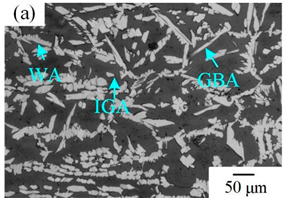	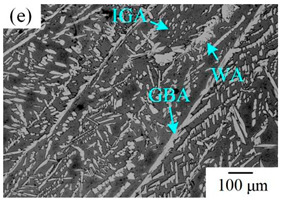
2.14	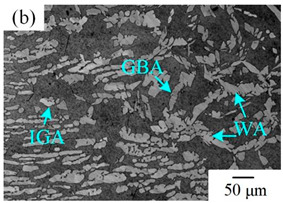	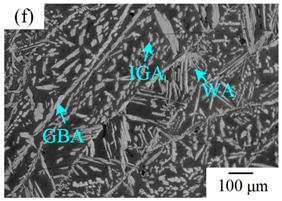
2.30	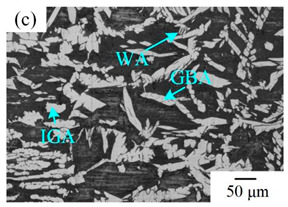	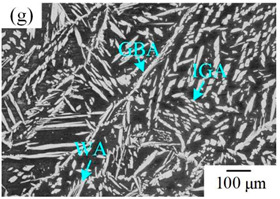
2.46	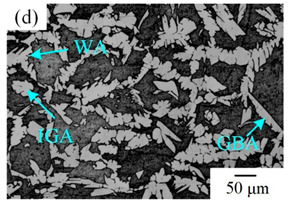	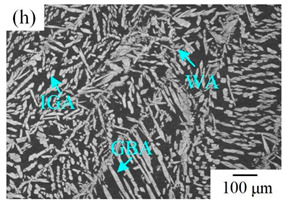

**Table 4:** The austenite content and chromium nitride precipitates in the HAZ (1–8) and WMZ (9–11).

Heat input (kJ/mm)	Austenite content (%)		Chromium nitride precipitates 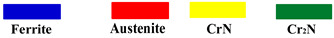	
	HAZ	WMZ	HAZ	WMZ
1.99	33.9	36.5	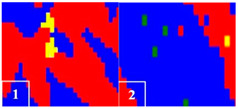	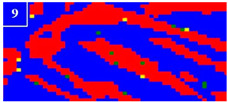
2.14	35.8	38.9	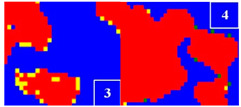	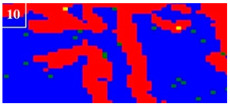
2.30	36.4	39.4	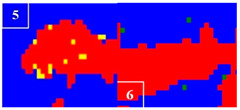	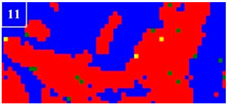
2.46	37.1	41.8	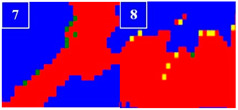	No chromium nitride precipitates were detected
The positions of CrN and Cr_2_N			In the ferrite, in the austenite, ferrite/austenite interfaces	In the ferrite, in the austenite, ferrite/austenite interfaces

**Table 5:** Corrosion characteristics of the HAZ and WMZ of the WJs in a 1mol/L NaCl solution at room temperature.

		E_corr_ (mV)	E_P_ (mV)	E_P_ − E_corr_ (mV)	I_corr_ (log(A/cm^2^))
HAZ	1.99 kJ/mm	−340 ± 12	155 ± 13	495 ± 8	−6.00 ± 0.30
	2.14 kJ/mm	−335 ± 20	178 ± 9	513 ± 10	−5.92 ± 0.07
	2.30 kJ/mm	−328 ± 25	205 ± 4	533 ± 10	−5.99 ± 0.25
	2.46 kJ/mm	−312 ± 15	294 ± 5	605 ± 7	−6.15 ± 0.29
WMZ	1.99 kJ/mm	−321 ± 23	309 ± 12	630 ± 12	−6.17 ± 0.09
	2.14 kJ/mm	−312 ± 12	357 ± 7	669 ± 6	−6.16 ± 0.22
	2.30 kJ/mm	−300 ± 9	372 ± 22	672 ± 10	−6.29 ± 0.12
	2.46 kJ/mm	−230 ± 30	437 ± 23	667 ± 17	−6.68 ± 0.02

## Data Availability

Not applicable.
